# Microplastics Decrease the Toxicity of ^137^Cs in the Zebrafish Embryo-Larva

**DOI:** 10.3390/toxics14040343

**Published:** 2026-04-20

**Authors:** Fangni Du, Wenjun Zhao, Shaofei Cao, Rui Zhang, Yuchen Yin

**Affiliations:** 1 China Institute for Radiation Protection, Taiyuan 030006, China; dufangni@cirp.org.cn (F.D.); zhangrui@cirp.org.cn (R.Z.); yinyuchen200076@163.com (Y.Y.); 2 Key Laboratory of Radiation Environment & Health of the Ministry of Ecology and Environment, Taiyuan 030006, China; 3 CNNC Key Laboratory for Radiation Protection Technology, Taiyuan 030006, China; 4 State Key Laboratory of Estuarine and Coastal Research, East China Normal University, Shanghai 200241, China; 52263904025@stu.ecnu.edu.cn

**Keywords:** ^137^Cs, microplastics, zebrafish embryos, toxicity, ecological risks

## Abstract

Large amounts of radionuclides and microplastics (MPs) have been released and will continue to be discharge into the environment. They will exist and interact in the aquatic environment over extended periods. However, the toxicological risks associated with their co-exposure remain poorly understood. In this study, the zebrafish (*Danio rerio*) embryos were exposed to ^137^Cs (6.8 × 10^4^ Bq/L) in combination with 9.9 μm polystyrene MPs (PS-MPs, 10, 100 μg/L) for 7 days. Early developmental growth was significantly influenced in the ^137^Cs-exposed groups. This was evidenced by delayed hatching, increased swimming total distance, and anxiety behavior (increasing swimming distance in the inner circle). Furthermore, transcriptomic analysis demonstrated that a higher number of differentially expressed genes were found in the ^137^Cs group compared to other exposure groups. In ^137^Cs groups, KEGG enrichment analysis highlighted significant disruptions in lipid metabolism pathways. ^137^Cs can influence its neuro-related genes by inducing lipid metabolism toxicity, providing a mechanistic explanation for the observed locomotory abnormalities in larvae. Interestingly, during the early stage of development, MPs appeared to reduce the internal irradiation dose and toxic effect by absorbing the ^137^Cs. Overall, this study enhances our understanding of the ecological risks posed by combined exposure to ^137^Cs and MPs.

## 1. Introduction

Nuclear power, recognized for its low carbon emissions and high energy efficiency, is expected to play an increasingly important role in future energy development. Under routine operation, nuclear power plants may discharge wastewater containing radionuclides into the surrounding environment. In particular, nuclear accidents, such as the Fukushima Daiichi disaster, have led to the release of large amounts of radioactive nuclides (^137^Cs, ^131^I, and ^90^Sr) into the marine environment [1]. Even after such events, more than 1 million tons of radioactive water are projected to be released into the ocean over the next 30 years [2]. Previous studies have shown that ionizing radiation can cause DNA damage, alter enzymatic activity, and cause behavioral changes or even developmental impairments in fish [3,4,5]. Moreover, certain radionuclides can adsorb onto environmental particulates, forming composite pollutants that may exert enhanced toxic effects on organisms [6,7].

Microplastics (MPs, <5 mm in size) have emerged as a novel class of contaminants and a global environmental concern [8]. As a major sink for pollutants, the ocean has been contaminated by MPs for long periods. Widespread occurrence of MPs across diverse marine environments, from coastal zones and open oceans to deep-sea trenches [9,10]. This ubiquity elevates the exposure risk for marine organisms, including fish. Due to their large specific surface area and hydrophobic properties, MPs readily adsorb other pollutants and serve as carriers for contaminant transport [11]. After ingestion, such composite pollutants can cause intestinal damage and induce synergistic toxic effects, thereby amplifying biological harm [12,13]. However, the previous studies have not focused on the comprehensive toxic effects of MPs and radioactive nuclides.

^137^Cs is one of the most representative and ecologically significant artificial radionuclides. It has a relatively long half-life (30.2 years) and can release β and γ rays. ^137^Cs can also adsorb to particulates and readily bioaccumulate in organisms [14]. Consequently, it has become a key target in marine radioactivity monitoring. Early life stages of organisms are generally more sensitive to radioactive and other environmental stressors than adults. Disruptions during development may have profound and long-term impacts on individual health and survival [15,16].

In this study, the ^137^Cs and polystyrene (PS) microbeads were selected as representative pollutants. Zebrafish (*Danio rerio*), a well-established model organism, was used to investigate the combined effects of ^137^Cs and MPs on embryonic and larval development. Exposure experiments were conducted using both high and low concentrations of MPs in combination with ^137^Cs. Developmental parameters and swimming behaviors were assessed, and transcriptomic analysis was conducted to elucidate the molecular mechanisms underlying the observed toxicity. The results of this study are intended to provide a scientific foundation for ecological risk assessment of ^137^Cs-MPs composite pollution.

## 2. Materials and Methods

### 2.1. Experimental Setup of MPs and ^137^Cs

The concentration of ^137^Cs was set as 6.8 × 10^4^ Bq/L based on reported accident levels [17]. The green fluorescence-labeled polystyrene (PS) MPs with a diameter of 9.9 μm were purchased from Thermo Fisher Scientific Inc. (Waltham, MA, USA). The exposed high and low concentrations of PS-MPs were 100 and 10 μg/L in the presence of 6.8 × 10^4^ Bq/L ^137^Cs. Scanning Electron Microscopy (SEM) (GeminiSEM450, Zeiss, Oberkochen, Germany) was used to acquire high-quality images of PS-MPs. Twenty μL of unprocessed dilution PS solution and experimental exposure PS solution were, respectively, transferred to the surface of the silicon wafer using a pipette. PS-MPs on the silicon wafer were imaged using SEM with various magnifications (828–3.55k×). Surface elemental composition was further characterized by energy-dispersive X-ray spectroscopy (EDS) (Zeiss, Oberkochen, Germany). The experiment setup was divided into 3 treatment groups and 1 control group.

To investigate whether PS-MPs adsorb ^137^Cs, a high-concentration solution was prepared containing 5.5 mg/L PS-MPs in 6.8 × 10^4^ Bq/L ^137^Cs. The mixture was homogenized and stood for 48 h. Then the MPs were removed by filtration through a 0.45 μm membrane filter, and the radioactivity of the filtrate was measured using a high-purity germanium (HPGe) gamma spectrometer (GEM-SP8530P4-RB, ORTEC, Oak Ridge, TN, USA).

### 2.2. Zebrafish Maintenance

All experimental procedures involving fish were conducted in accordance with the Guidelines for the Ethical Review of Laboratory Animal Welfare (People’s Republic of China National Standard GB/T 35892-2018) and OECD guideline standard (No.177) [18,19]. This study was approved by the Ethics Committee of the China Institute of Radiation Protection (Approval No. 20250701). Adult zebrafish (AB strain, 6–10 months old), including ten females and twenty males, were purchased from the Institute of Hydrobiology (Chinese Academy of Sciences) and placed in a tank containing 30 L filter water. One-third of the water in the fish tank was renewed twice per week, and fish were fed *Artemia salina* twice a day. The rearing conditions were maintained at a 14 h light/10 h dark photoperiod, a temperature of 27–28 °C, and a pH of approximately 7.5.

### 2.3. Embryo Collection

To obtain embryos, adult female and male zebrafish were paired at a ratio of 1:2 and placed in ten spawning tanks at 17:00. Spawning was induced by the onset of light at 08:00 the following morning. After 1 h of illumination, fertilized embryos deposited at the bottom of the tanks were collected using a Pasteur pipette. Embryos were rinsed with embryo medium and examined under a stereomicroscope (SZ680, CNOPTEC, Chongqing, China) within 2 h post-fertilization (hpf) to select normally developing embryos. Embryo exposure experiments were conducted from 3 to 168 hpf. For the developmental toxicity assay, embryos were randomly exposed in 6-well plates (*n* = 6 replicates), with 10 embryos per well and 10 mL of exposure solution per well. During the exposure period, embryos were maintained in an incubator characterized (SPX-250LF-F, Shanghai Jingqi Instrument Co., Ltd., Shanghai, China) by uniform illumination and stable temperature. For gene expression analysis, embryos were exposed in 9 cm Petri dishes (*n* = 6 replicates), each containing 50 embryos in 50 mL of exposure solution. All experiments were conducted under semi-static conditions, with half of the exposure medium renewed every 24 h.

### 2.4. Developmental Toxicity

Developmental toxicity data were obtained from 10 embryos-larvae per well in 6-well plates at multiple time points (*n* = 6 replicates). Survival was assessed at 168 hpf, and hatching rate was recorded at 72 hpf. Survival and hatching rates were calculated as the proportion of living embryos or hatched larvae relative to the total number of fertilized eggs. Heart rate was measured at 48 hpf by counting beats over a 15 s interval under a light microscope. Morphological abnormalities were evaluated at 96 hpf using a light microscope after placing larvae on a hanging-drop slide. The incidence and types of malformations were determined by counting the number of malformed larvae relative to the total number of larvae examined.

### 2.5. Larvae Swimming Behavior

The swimming behavior of zebrafish larvae at 168 hpf was evaluated to assess ^137^Cs-MPs-induced metabolism and neurobehavioral alterations. Larvae without visible malformations (e.g., spinal curvature, pericardial edema, or yolk sac edema) were randomly selected from each treatment group and individually recorded for 5 min in 12-well plates (one larva per well) following a 5 min acclimation period. The swimming trajectories and behavioral parameters were quantified using idtracker.ai (v5.2.12), with a total of eight larvae analyzed per treatment [20]. The parameters compared among exposure groups included total swimming distance, spatial distribution, mean acceleration, and mean turning angle.

### 2.6. Transcriptome and Quantitative PCR Analysis

To evaluate the effects of ^137^Cs and MPs on zebrafish embryonic development at the transcriptional level, the 90 larvae (*n* = 3 replicates) at 168 hpf were anesthetized using benzocaine and placed in a centrifuge tube. They were transported on dry ice to a commercial transcriptome analysis company (Shanghai Majorbio Technology Co., Ltd., Shanghai, China) on dry ice for RNA-seq and quantitative PCR (qPCR) analyses. The RNA-seq analysis was performed on the Majorbio platform (https://v.majorbio.com/project-center/overview, accessed on 7 April 2026). Personnel performing transcriptome analysis and qPCR were blinded to the group allocation. The differentially expressed genes (DEGs) were identified by the criteria that fold change ≥ 2 and adjusted *p*-value < 0.05. Functional annotation of DEGs was conducted using the Kyoto Encyclopedia of Genes and Genomes (KEGG) database, with pathways considered significantly enriched at *p* < 0.05. To validate transcriptomic results, the expression levels of 12 representative DEGs related to lipid metabolism and energy homeostasis, neurotransmission and neuromodulation, together with a reference gene, were quantified by qPCR. Primer sequences and detailed bioinformatic methods are provided in the Supporting Information (Table S1).

### 2.7. Estimation of Internal and External Dose

Considering the small body size of the larvae, large uncertainties were associated with the measurement of adsorbed ^137^Cs activity. Therefore, the activity was estimated using the reported adsorption coefficient for zebrafish, in combination with the water volume and radionuclide activity [21]. According to the exposure concentration, water volumes and the concentration factor of ^137^Cs, Monte Carlo simulations were used to estimate internal and external dose rates for zebrafish under both laboratory and environmentally relevant scenarios.

### 2.8. Statistical Analysis

Statistical analyses were conducted using Python (version 3.13.7). The data (developmental, swimming behavior) were involved in the analysis. Data normality was evaluated using the Shapiro–Wilk test. As the data were not normally distributed, group differences were assessed using the Kruskal–Wallis test, followed by Dunn’s post hoc test with Bonferroni correction for multiple comparisons. Statistical significance was set at *p* < 0.05.

## 3. Results and Discussion

### 3.1. Characterization and Adsorption of ^137^Cs on PS-MPs

**Figure 1:**
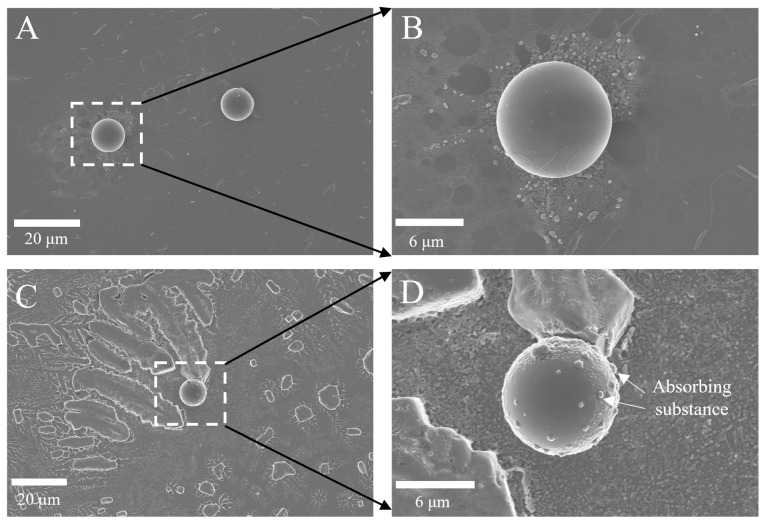
The SEM image of MPs. (**A**,**B**), primary particles; (**C**,**D**), culture medium soaked.

SEM provided high-resolution images of the PS-MPs. The pristine particles exhibited smooth surfaces with no visible attachments (Figure 1A,B). After 48 h incubation in culture medium containing ^137^Cs, noticeable deposition of materials from the medium was observed on the particle surfaces (Figure 1C,D). EDS analysis confirmed the presence of adsorbed inorganic ions, including Fe, on the PS-MPs (Figure S1), indicating that components of the medium readily associate with the particle surface. The activity of ^137^Cs associated with individual PS-MPs was estimated to be 2 mBq per particle. Similar adsorption behavior has been reported for other polymer types, including high-density polyethylene (HDPE) and polyvinyl chloride (PVC) [22]. Previous studies further demonstrate that adsorption characteristics differ between pristine and environmentally weathered MPs [23,24].

### 3.2. ^137^Cs and PS-MPs Induce Developmental Toxicity of Embryonic Zebrafish

**Figure 2:**
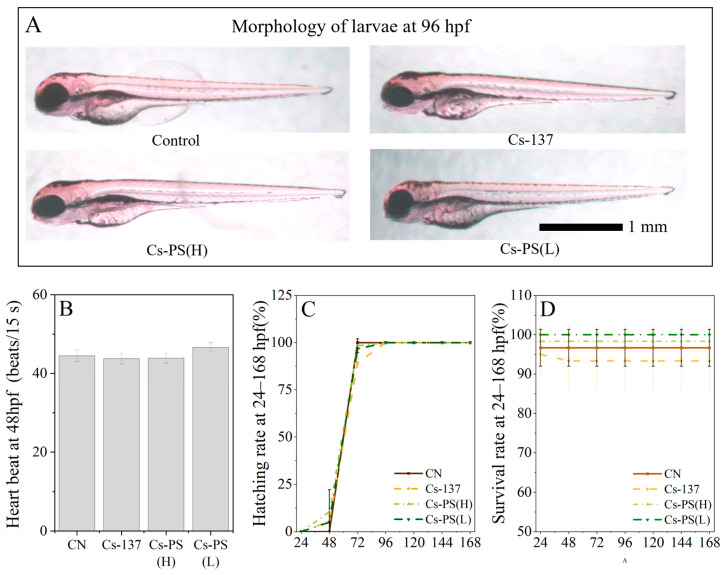
Effects of PS and ^137^Cs exposure on developmental toxicity of zebrafish embryos. (**A**), morphology; (**B**), heart beats; (**C**), hatching rate; (**D**), survival rate.

No obvious morphological abnormalities were observed in embryos or larvae following short-term exposure. Representative larval morphology is shown in Figure 2A, and body length did not differ significantly among the four groups. At 72 hpf, the hatching rate values were 100, 89 ± 0.9, 98 ± 3.7, and 97 ± 4.7, respectively. The hatching rate was significantly reduced in the ^137^Cs-only group compared with the control (Figure 2C). Survival rates ranged from 93.3% to 100% across all treatments, with no significant differences observed. These results indicate that exposure to ^137^Cs alone can delay embryonic development, as reflected by reduced hatching success. However, the presence of PS-MPs appeared to attenuate this developmental toxicity in a concentration-dependent manner.

Hatching represents a critical life-history state that directly influences larval survival, feeding, and subsequent development. At 72 h post-fertilization, the physical and morphological development of zebrafish is essentially complete, following which hatching out of the membrane occurs. In the present study, we demonstrate that ^137^Cs exposure significantly delayed hatching, which is consistent with previous reports on radionuclide-induced developmental toxicity [5]. Notably, co-exposure to PS-MPs mitigated the inhibitory effect of ^137^Cs on hatching.

### 3.3. ^137^Cs and PS-MPs Induce Behavior Changes in Zebrafish Larvae

**Figure 3:**
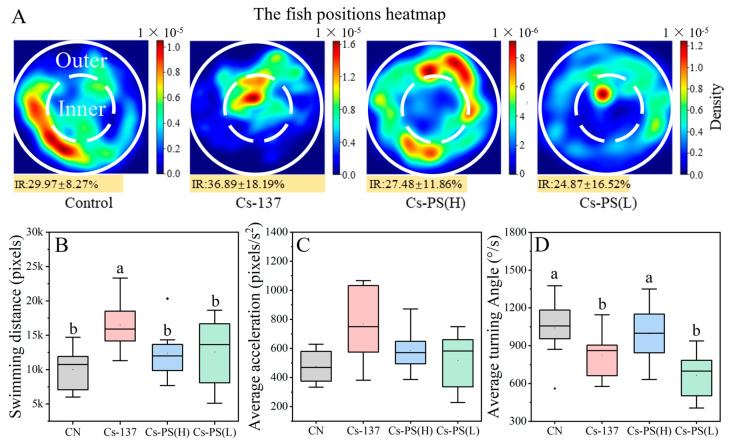
Effects of PS and ^137^Cs exposure on behavior of zebrafish larvae. (**A**), the heat map of larvae between the inner and outer circles (Inner Ratio, IR); (**B**), the total swimming distance of larvae; (**C**), the average acceleration of larvae; (**D**), the total turning angle of larvae. Note: Different superscript letters (a, b) denote statistically significant differences between the two datasets (*p* < 0.05).

Locomotor activity is a sensitive behavioral endpoint in zebrafish. Heat map analysis showed reduced time spent in peripheral zones and increased occupancy of the central area in the ^137^Cs-exposed group (Figure 3A). Consistent with this spatial redistribution, larvae in the ^137^Cs group exhibited a significantly greater total swimming distance compared with the other treatment groups (Figure 3B). In addition, the mean turning angle was significantly reduced in both the ^137^Cs group and the low-concentration PS-MPs co-exposure group (Figure 3D).

Swimming behavior in zebrafish is also a critical endpoint for evaluating the toxicity of environmental pollutants. In previous studies, low-dose gamma radiation has been shown to stimulate larval activity, resulting in increased swimming speed, whereas higher doses beyond a threshold suppress locomotion [5,25]. In the present study, exposure to ^137^Cs similarly induced hyperactivity, as evidenced by increased swimming distance. Trajectory analysis further indicated that control larvae rapidly explored and adapted to the novel environment, whereas ^137^Cs-exposed larvae exhibited altered spatial exploration patterns. Specifically, larvae preferentially occupied the central region rather than the periphery, which is commonly interpreted as an anxiety-like behavioral phenotype [26].

Previous studies have reported that exposure to MPs at concentrations below 100 μg/L does not induce pronounced alterations in locomotor behavior [27,28]. This suggests that PS-MPs alone exert minimal effects on zebrafish behavior under the exposure conditions used in this study. However, co-exposure to PS-MPs partially mitigated ^137^Cs-induced behavioral abnormalities in larvae. This pattern is consistent with the developmental toxicity outcomes described in Section 3.2.

### 3.4. ^137^Cs and PS-MPs Induce Alterations in the Expression of Genes

**Figure 4:**
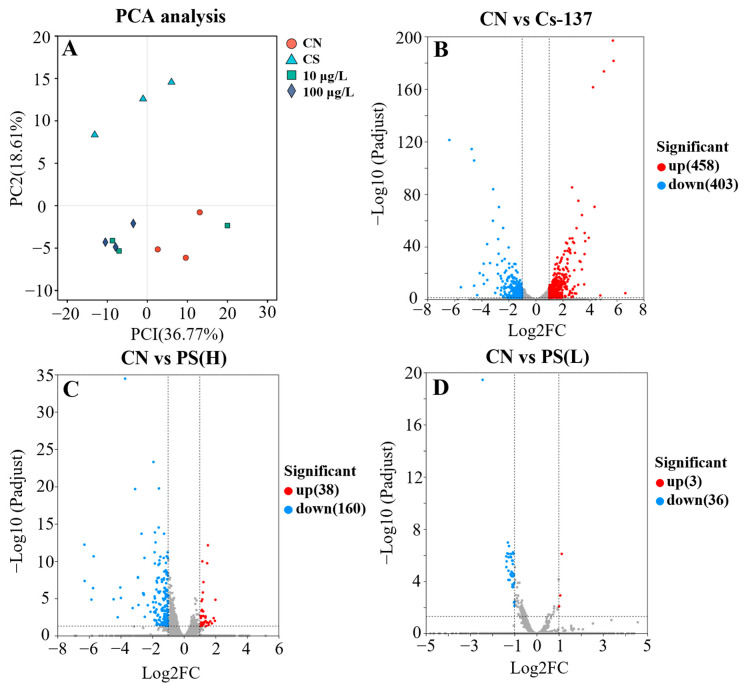
RNA-Seq analysis of zebrafish larvae at 168 hpf. (**A**), Principal components analysis (PCA); (**B**–**D**), Volcanic diagrams comparing different groups.

**Figure 5:**
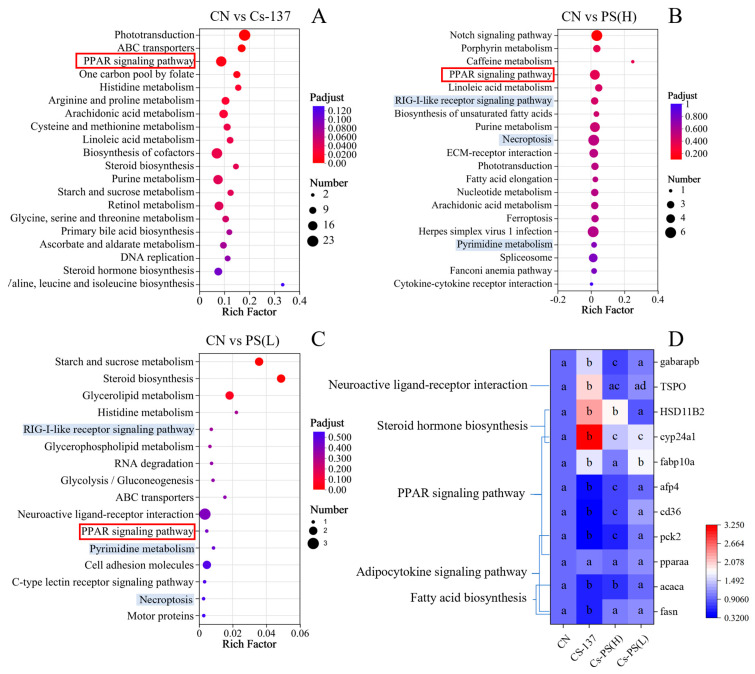
RNA-Seq analysis of zebrafish larvae at 168 hpf. (**A**), Principal components analysis (PCA); (**B**–**D**), Volcanic diagrams comparing different groups. KEGG pathway enrichment and qPCR validation results. (**A**–**C**) The top 20 KEGG pathways enriched in the ^137^Cs, Cs-PS(H) 100  μg/L and Cs-PS(L) 10 μg/L exposure groups, respectively; (**D**), the gene expression in signal transduction and lipid metabolism. Note: Different superscript letters (a, b, c, d) denote statistically significant differences between the two datasets (*p* < 0.05). Red boxes: pathways detected in all three groups. Blue background: pathways detected in two groups.

PCA revealed clear separation between the control and exposure groups (Figure 4A). The first two principal components (PC1 and PC2) explained 36.77% and 18.61% of the total variance, respectively (Figure 4B). The ^137^Cs-exposed group was distinctly separated not only from the control group but also from the PS-MPs-exposed groups. The two PS-MPs concentration groups exhibited high similarity to each other. A total of 861, 198, and 39 DEGs were identified in the ^137^Cs, PS-MPs 100 μg/L, and PS-MPs 10 μg/L groups, respectively (Figure 5B–D). The number of DEGs in the ^137^Cs group was substantially higher than in the other groups, indicating a stronger transcriptional perturbation. Three common DEGs were identified in both the ^137^Cs and ^137^Cs + PS-MPs co-exposure groups, including *per2*, *myhc4*, and *ctrl* (Figure S2).

Among the top 20 enriched KEGG pathways, the PPAR signaling pathway was shared across all treatment groups (Figure 5). Functional enrichment analysis showed that DEGs in the ^137^Cs group were primarily associated with signal transduction, lipid metabolism, and amino acid metabolism (Figure 5A). In contrast, the two PS-MPs exposure groups exhibited more similar enrichment profiles, including pathways such as RIG-I-like receptor signaling, necroptosis, and pyrimidine metabolism (Figure 5B,C). Overall, PS-MPs exposure predominantly affected pathways related to signal transduction and signaling molecule interactions.

To validate the transcriptomic results, representative genes were selected for qPCR verification (Figure 5D). Genes involved in the PPAR signaling pathway (*fabp10a*, *afp4*, *cd36*, and *pck2*) and fatty acid biosynthesis (*acaca* and *fasn*) were significantly downregulated in the ^137^Cs-exposed group. Conversely, ^137^Cs significantly upregulated the expression of genes involved in neuroactive ligand–receptor interaction, steroid hormone biosynthesis, and retinol metabolism, including *tspo*, *hsd11b2*, and *cyp24a1*. The qPCR results were largely consistent with RNA-seq data, confirming the reliability of the transcriptomic analysis.

Previous studies have shown that disruption of *GABA* signaling, the principal inhibitory neurotransmitter system in the vertebrate brain, is closely associated with anxiety-related behaviors [29]. *TSPO* is involved in neurosteroidogenesis, and its upregulation has been proposed as a sensitive biomarker of neuroinflammation and neural injury [30,31]. Consistent with these findings, the upregulation of neuro-related genes such as *gabarapb* and *tspo* observed in this study suggests that ^137^Cs-induced behavioral abnormalities are associated with alterations in neurotransmission and neuroinflammatory processes.

Both RNA-seq and qPCR results further demonstrated that ^137^Cs exposure significantly altered multiple metabolic pathways. Altered expression of *hsd11b2* and *cyp24a1* indicates disruption of steroid metabolism, and differential regulation of *hsd11b2* has previously been linked to behavioral alterations [32]. Moreover, ^137^Cs suppressed multiple genes within the PPAR signaling pathway and lipid metabolism pathways, suggesting an inhibitory effect on lipid metabolic processes. Consistent with previous toxicological studies, chronic acrylamide exposure has been shown to disrupt lipid and fatty acid metabolism in zebrafish, leading to anxiety- and depression-like behaviors [33].

Disruption of lipid metabolism may exert broader effects on neural development and function. Lipids are critical components of neuronal membranes and myelin, and their dysregulation has been implicated in neurodegenerative disorders, including Parkinson’s, Alzheimer’s, and Huntington’s diseases [34,35]. In zebrafish, exposure to diisononyl cyclohexane-1,2-dicarboxylate (*DINCH*) has been shown to disrupt lipid metabolism and impair neurodevelopment [36]. Similarly, perfluorinated compounds such as PFBA and PFAS have been reported to alter lipid homeostasis and induce neurobehavioral toxicity [37]. Collectively, these findings support the hypothesis that, beyond direct effects on neurotransmission, ^137^Cs-induced disruption of lipid metabolism may contribute to the observed behavioral abnormalities by interfering with neural development and neurotransmitter systems. In this study, the transcriptomic results provided only preliminary insights into the potential mechanisms underlying the effects of combined pollutants on larvae. The full mechanisms remain to be further clarified. For example, targeted assays, such as reactive oxygen species (ROS) measurement and DNA double-strand break (DSB) detection, are required to validate the proposed pathways.

### 3.5. Underestimation of Toxic Effects of ^137^Cs and PS-MPs in Realistic Environmental Conditions

**Figure 6:**
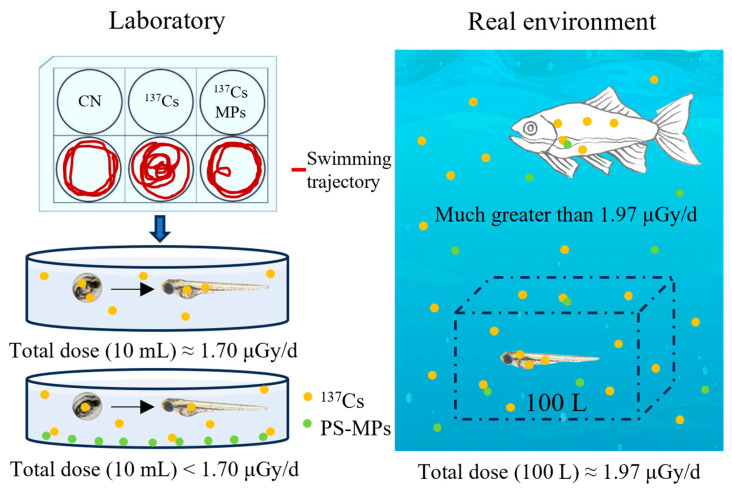
Underestimation of the toxicity of ^137^Cs-MPs in real environment.

During the seven-day developmental period, zebrafish larvae through branchial uptake continuously absorbed and accumulated ^137^Cs from the surrounding water. Based on the equilibrium concentration factor of ^137^Cs in zebrafish and the exposure volume, the internal dose rate was 1.60 μGy/d, and external radiation dose rates for 10 mL and 100 L were 0.10 and 0.37 μGy/d, respectively (Figure 6). Comparison with previously reported experimental datasets indicates that the total radiation dose in this study was relatively low, suggesting that ^137^Cs can still induce measurable biological effects even at low exposure levels. In the presence of MPs, a proportion of ^137^Cs was adsorbed onto the particle surfaces. Because larvae at this developmental stage had not yet initiated exogenous feeding, oral ingestion of MPs was unlikely. Consequently, the adsorption of ^137^Cs onto MPs may have reduced the bioavailable fraction of the radionuclide in the water, thereby lowering internal dose and partially attenuating the observed biological effects. Although this conclusion is based on Monte Carlo modeling and theoretical estimations, and the actual radionuclide dose in the larvae has not been directly measured, the result offers a preliminary insight. It suggests that, under small-volume exposure conditions, MPs may exert an apparent short-term protective effect by reducing radionuclide bioavailability, which should be further validated experimentally.

However, radiation dose is strongly influenced by the volume of the exposure medium. Monte Carlo simulation suggests that when larvae are exposed to larger water volumes (e.g., 100 L), the cumulative radiation dose would increase compared with the present experimental conditions. In natural aquatic environments, the effective dose from ^137^Cs may be considerably higher. Moreover, the role of MPs may shift over time. While MPs may reduce radionuclide bioavailability during early developmental stages when ingestion does not occur. When the fish start to feed, MPs may act as vectors for ^137^Cs. It will provide an additional oral exposure pathway. Similar vector effects have been reported for other contaminants, including mercury, where MPs enhanced bioaccumulation and toxicity in adult fish [38]. This study still has experimental limitations. The present work focused on the combined effects under freshwater conditions. Seawater environments, which contain abundant ions such as Na^+^, K^+^, Mg^2+^, and Ca^2+^, were not considered. These ions may affect the adsorption behavior of Cs ions on MPs. Therefore, further studies are needed to investigate the toxic effects under more environmentally realistic conditions.

## 4. Conclusions

In summary, this study demonstrates that accident concentrations of ^137^Cs can delay embryonic hatching and alter locomotor behavior in zebrafish larvae. Transcriptomic analyses further indicate that ^137^Cs disrupts neural signaling pathways, potentially through modulation of lipid metabolism and neuroendocrine-related processes, ultimately contributing to behavioral abnormalities. Co-exposure with MPs reduced alterations in physiological, behavioral, and transcriptomic responses under the present experimental conditions. This effect is likely attributable to partial adsorption of ^137^Cs onto MPs surfaces, which reduced its bioavailability and internal radiation dose in larvae. Nevertheless, under environmentally realistic conditions, MPs may act as vectors for radionuclide transport and uptake. Therefore, interactions between radionuclides and MPs represent a complex and dynamic risk factor that cannot be overlooked in ecological risk assessment of radioactive and MPs pollution. In the future, studies are required to fully elucidate the combined impacts of ^137^Cs and MPs on aquatic organisms for the long-term.

## Data Availability

The original contributions presented in this study are included in the article. Further inquiries can be directed to the corresponding author.

## Supplementary Materials

The following supporting information can be downloaded at: https://www.mdpi.com/article/10.3390/toxics14040343/s1. Figure S1: Energy Disperse Spectroscopy (EDS) analyses of PS-MPs. Figure S2: Venn diagram of the overlapping relationship between gene sets. Table S1: PCR primers used for the gene expression analyses.

## Abbreviations

The following abbreviations are used in this manuscript:

MPs	Microplastics
PS	Polystyrene
HPGe	High-purity germanium
hpf	h post-fertilization
DEGs	The differentially expressed genes
KEGG	The Kyoto Encyclopedia of Genes and Genomes
HDPE	High-density polyethylene
PVC	Polyvinyl chloride
