# Supplementary material for: Microplastics Decrease the Toxicity of 137Cs in the Zebrafish Embryo-Larva
Source: Toxics. 2026 Apr 20;14(4):343. doi: 10.3390/toxics14040343 (PMC13120410; doi:10.3390/toxics14040343)
Supplement: Supplementary file 1 [file toxics-14-00343-s001.zip › toxics-4243527-supplementary.pdf]

Supplementary materials

Microplastics Decrease the Toxicity of ^{137}Cs in the Zebrafish Embryo-Larva

Fangni Du [1,2,3], Wenjun Zhao [4], Shaofei Cao [1,2,3,*], Rui Zhang [1,2,3] and Yuchen Yin [1,2,3]

[1] China Institute for Radiation Protection, Taiyuan 030006, China

[2] Key Laboratory of Radiation Environment & Health of the Ministry of Ecology and Environment, Taiyuan 030006, China

[3] CNNC Key Laboratory for Radiation Protection Technology, Taiyuan 030006, China

[4] State Key Laboratory of Estuarine and Coastal Research, East China Normal University, Shanghai 200241, China

* Correspondence: caoshaofei@cirp.org.cn

Legends of Supplementary Tables

Table S1. PCR primers used for the gene expression analyses.

Legends of Supplementary Figure

Figure S1. Energy Disperse Spectroscopy (EDS) analyses of PS-MPs.

Figure S2. Venn diagram of overlapping relationship between gene sets.

Table S1. PCR primers used for the gene expression analyses.

Genes name	Primer name	Primer sequence (5'-3')	GenBank No.
TSPO	Forward	AAGAGCTTGGAGGCTTCACC	NM_001006032.2
	Reverse	GCTCTATCAGTGCCAGTTGA	
HSD11B2	Forward	CAACACACTTCGTCACGAGC	NM_212720.2
	Reverse	GTTACTGCTCTGCCCTGTCTT	
gabarapb	Forward	CCAGACAGGGTTCCTGTAATTGT	NM_001386387.1
	Reverse	CCACAGTCAGATCAGAAGGGAC	
pparaa	Forward	CATGTCCCACAACGCTATTCG	NM_001161333.2
	Reverse	TGAGGTAGGCTTCGTGCATC	
afp4	Forward	CAACAAGGCCAATGCTTACC	NM_001045488.2
	Reverse	ATCTGCTCTTCAATGTTGCCG	
pck2	Forward	CTGGTTGGCTGAACACATGC	NM_213192.1
	Reverse	TGCTCTGAGTTTACCCTGACTG	
cd36	Forward	GCTGGCAAGGTGAAACATCAG	NM_001002363.1
	Reverse	TTCTTGTCCAGGAACGGGTG	
cyp24a1	Forward	GGCATGGATTTCATCACCGC	NM_001089458.1
	Reverse	GCTATTAGCCGTCGTCTCCA	
fabp10a	Forward	GGCAAGAAGCTCAAGTGCATCG	NM_152960.1
	Reverse	GCTCTTCCTGATCATGGTGGT	
acaca	Forward	TACATTGCTGCACTGGCTGT	NM_001271308.2
	Reverse	CCACGGCAACTACTCCAGTT	
fasn	Forward	CTCATTGGCGGAGTGAACCT	XM_009306806.5
	Reverse	GGAACGTCACACCTTGCTCT	
β-actin	Forward	AGCCTTCCTTCCTGGGTATGG	NM_131031.2
(control gene)	Reverse	TGGTACCTCCAGACAGCACT	

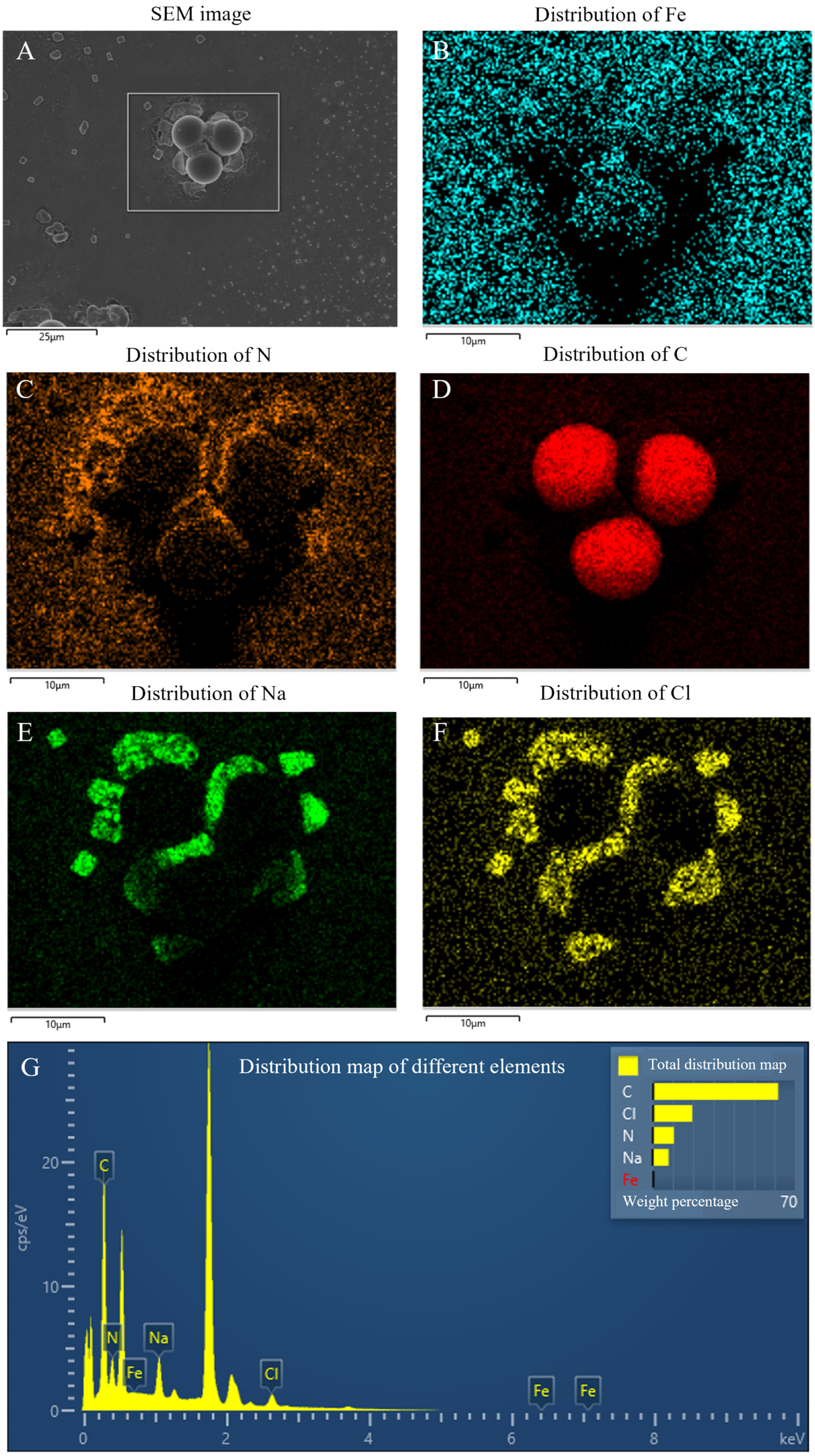

Figure S1. Energy Disperse Spectroscopy (EDS) analyses of PS-MPs.

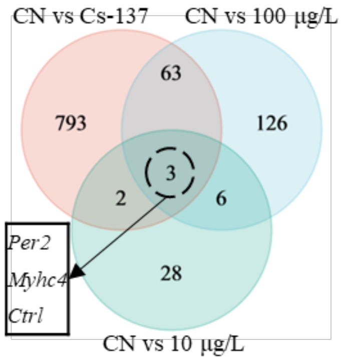

Figure S2. Venn diagram of overlapping relationship between gene sets.
